# Family interventions in dementia mental health environments (FIND ME): a mixed-methods protocol

**DOI:** 10.1136/bmjopen-2025-110244

**Published:** 2026-01-28

**Authors:** Emma Wolverson, Juliet Gillam, Rosie Dunn, Juanita Hoe, Benjamin R. Underwood, Shadreck Mwale, Nicki Credland, Martin Robertson, Megan Wyatt, Rhiannon Lane, Katie Featherstone

**Affiliations:** 1 Geller Institute of Ageing and Memory, University of West London, London, UK; 2 Old Age Psychiatry, Cambridgeshire and Peterborough NHS Foundation Trust, Cambridge, Cambridgeshire, UK; 3 Teesside University, Middlesbrough, England, UK; 4 Expert by Experience, University of West London, London, England, UK; 5 Department of Sociology, Philosophy and Anthropology, University of Exeter, Exeter, UK

**Keywords:** Dementia, MENTAL HEALTH, Family, Inpatients

## Abstract

**Introduction:**

Admission to a mental health ward can be distressing for people living with dementia and their carers. While carer involvement is associated with improved outcomes, carers often report feeling excluded from decision-making and support during admissions. There is limited understanding of how wards engage with carers and what strategies might enhance involvement. This study seeks to address this gap by exploring carer and patient experiences during and after admission and co-producing evidence-based strategies for improved support and involvement.

**Methods and analysis:**

The FIND ME study uses a convergent parallel mixed-methods design. A national online survey will map provision of mental healthcare for people with dementia across the UK. Narrative interviews will be undertaken with carers of current inpatients (n=24) and recently discharged individuals (n=24), with opportunities for dyadic interviews with people with dementia. Ethnographic fieldwork across three wards (30 days per site) will provide insight into organisational cultures, staff practices and carer involvement. Evidence-based co-design workshops with carers, people with dementia and staff will use these findings to identify priorities and develop practical strategies for service improvement. Finally, a feasibility study will test the acceptability, relevance and potential for implementation of these co-designed strategies. Quantitative data will be analysed descriptively, while qualitative data will undergo narrative and thematic analysis. Triangulation across datasets will ensure rigour.

**Ethics and dissemination:**

Ethical approval has been granted by London Camberwell St Giles Research Ethics Committee and the Health Research Authority (REC Ref: 25/LO/0040). Informed consent will be obtained from all participants, with capacity assessed in line with the Mental Capacity Act (2005). Dissemination will include peer-reviewed publications, conference presentations and freely available multilingual resources for carers, people with dementia and ward staff, supported by NHS and third-sector partners.

**Trial registration number:**

NIHR161439

**STRENGTHS AND LIMITATIONS OF THIS STUDY**

- A rigorous mixed-methods design (survey, longitudinal narrative interviews, ethnography, co-design and feasibility testing) to comprehensively examine carer involvement in dementia mental health wards.
- Strong integration of patient and public involvement with carers and people living with dementia shaping the study design, materials and data collection methods to ensure relevance and acceptability.
- Longitudinal narrative interviews allow for in-depth exploration of changing carer needs and experiences over time, but attrition may occur due to the length and sensitivity of follow-up.
- Ethnographic fieldwork across diverse ward settings (rural, inner-city and coastal) provides rich and varied contextual insights.
- The use of inclusive creative methods and multiple recruitment pathways strengthens representation of diverse carers, though despite best efforts some groups (eg, ethnic minority carers, very advanced dementia patients) may remain underrepresented.

## Introduction

 Care needs for people with dementia advance as the condition progresses.^1^ Often with dementia, the person’s ability to communicate verbally is compromised, so ‘symptoms’ may go undetected and unmet. Unmanaged symptoms and discomfort often manifest as changes in behaviour and mood, termed ‘neuropsychiatric’ symptoms or behavioural and psychological symptoms of dementia including agitation, anxiety and depression.^2^ Changes in behaviour and mood worsen over time if needs remain unmet.^2 3^ In the UK, in severe cases, admission to a mental health ward might be necessary if people are acutely unwell and features of their dementia cannot be managed at home or in other settings.^4^ Individuals are most frequently admitted to mitigate risks or dangers posed to the safety of the person living with dementia and those around them.

The average length of admission to these wards is 100 days, and most individuals with dementia will not return home.^5^ There is some variation between different National Health Service (NHS) trusts and health boards. Some provide care for this population in mixed wards alongside individuals with other mental health conditions such as depression, while others have specialist wards solely for patients living with dementia, undergoing assessment and treatment for mental health difficulties. Admission to specialist wards is advocated by the Royal College of Psychiatrists for this population if admission is possible.^4^

This vulnerable population is often in the advanced stages of dementia, and multimorbidity and frailty are common.^6^ Patients present with complex needs, which require person-centred and individualised care.^7^ This can be facilitated by promoting the involvement of carers, broadly defined as relatives, friends or partners of the person with dementia who provide care without getting payment. Previous research with other patient groups has demonstrated that sufficient carer involvement directly improves outcomes for patients residing on mental health wards. Family interventions—structured, therapeutic programmes designed to improve communication, coping and collaboration within families—are associated with better symptom control and improved functioning for adults with mental health conditions,^8^ reduced relapse rates, lower hospital readmissions and improved medication adherence—all key determinants of long-term stability.^9 10^ In addition, the National Institute for Clinical Excellence highlights that supported and informed carers contribute to earlier detection of deterioration, improved engagement with treatment and more consistent, coordinated care for patients.^11^

Family carers have unique insight into their friend’s or relative’s history, needs and care preferences, such as their routines and methods of communication, elements of which ward staff may not fully understand.^12^ Their involvement is important, to ensure care delivery is appropriate and centred around the specific needs of the individual. Promoting shared decision-making is integral to good quality dementia care^12 13^ and is of particular importance when a patient might not be able to advocate for themselves or lacks the capacity to make decisions concerning their own care and treatment.^14^

Despite this, carers are often inadequately involved in the care of their friend or relative on admission to a mental health ward. This issue was amplified by the COVID-19 pandemic, which added an unprecedented challenge to an already complex and delicate relationship between staff and carers.^15^

Carers report feeling marginalised and excluded from care decisions around treatment options and discharge planning, despite their extensive knowledge of their friend or relative’s needs.^16^ They may sometimes experience what they feel is a distinct lack of communication following admission, with fragmented and insufficient information sharing, and a sense of feeling like an ‘outsider’, with limited visiting opportunities and challenges around navigating the legal frameworks surrounding the admission.^17^

Mental health admissions can have serious detrimental effects on caregiver well-being, with increased exhaustion and depression.^18 19^ Carers are not routinely offered emotional or practical support.^20^ This intensifies an already acutely distressing period for them, with reports of an absence of processes in place to ameliorate their concerns and help cope with the emotional strain of their situation.^17^

### Evidence gap

This study responds to calls from NHS England and the Department of Health and Social Care for collaborative projects using participatory co-production approaches, to actively involve carers in the planning, development and evaluation of services^21^ and work in partnership with carers and service users,^22^ to ensure interventions are responsive to user needs and guided by the needs of those who will receive and deliver care.^23^

The importance and benefits of involving key stakeholders in the development of research is becoming increasingly recognised to strengthen its success, in regard to both outcomes and implementation.^24^ Participatory research necessitates a shift in the traditional power balance between researchers and end-users to ensure collective ownership, equal participation and legitimate shared decision-making.^25^ It has also been associated with facilitating the delivery of person-centred care.^26^ Early involvement of end-users in research design is a key strategy for promoting translation of findings into practice and is of particular importance in dementia research, where people with dementia themselves have traditionally been neglected.^27^

### Aims and objectives

The aim of this study is to improve our understanding of carer experiences when a person living with dementia is admitted to a mental health ward. We will look at the support needs of carers, and how these are understood and recognised by staff. We will seek to understand ward staff rationales behind their responses to carers, and how carers are involved in decisions regarding care from admission, during their stay and following discharge. We will develop evidence-based strategies with the aim of ensuring that mental health wards can better support and cooperate with carers to improve carer experience and to improve patient outcomes and the care which people with dementia receive on a mental health ward.

### Objectives

1. To describe current staffing structures, patient profiles and existing support mechanisms for carers and families in mental health wards for people living with dementia in the UK.
2. To understand carer experiences, perspectives on their involvement and support needs throughout the trajectory of an admission of their friend, partner or relative living with dementia to a mental health ward, from time of admission to post-discharge.
3. To examine (a) routine practice on the ward which includes or excludes carers, (b) how staff recognise and respond to carers’ needs and (c) any formal frameworks and informal rationales which impact staff’s involvement or exclusion of carers from decisions regarding care, to inform our understanding of organisational cultures and staff perspectives on mental health wards.
4. To co-design evidence-based strategies using the findings to facilitate carer involvement and support and promote best practice.
5. To assess the feasibility of implementing the strategies in practice.

## Methods and analysis

The theoretical underpinnings and key paradigms which are integral to the FIND ME study are presented first, followed by a detailed description of the planned research methods.

### Underpinning theory

The study is underpinned by family systems theory and the Family Adjustment and Adaptation Response (FAAR) model,^28 29^ which offers a framework for examining how families respond to and manage stressors, such as dementia and mental health-related hospital admissions. The FAAR model conceptualises family functioning as a dynamic process in which families strive to balance demands (eg, illness-related stress) with their available capabilities (eg, resources, social support and coping strategies) to enable them to adjust and adapt over time. It emphasises both the structural and interactional aspects of family life, recognising how families reorganise roles, relationships and routines in response to ongoing challenges.

Complementing this, theoretical perspectives from anthropology and sociology are drawn on to explore culturally and socially embedded understandings of family and kinship within care contexts, as well as how healthcare professionals and mental health workers recognise and engage with families. This integrated theoretical approach seeks to generate a nuanced understanding of carer and family experience, with the aim of identifying effective strategies to support them during mental health ward admissions.

### Involvement of people with dementia and their carers

Patient and public involvement (PPI) is a vital component of the FIND ME study and has been incorporated rigorously from its inception. Our PPI advisory group—The Inpatient Dementia Experience Group—comprises eight individuals: one living with dementia and seven current and bereaved carers. All individuals have experience of inpatient mental health admissions. Two of the study’s co-applicants also have relevant lived experience: one living with dementia and one bereaved carer.

The impetus to conduct this study was driven by the group highlighting the lack of carer support as a priority. The group has been crucial in developing the research proposal, the design of the study components, writing the ethics application and in the development of all patient-facing documents. We meet monthly to discuss how the study is progressing and to seek their input and ensure their continued involvement. This will continue throughout the study.

### Study design

We will employ a convergent parallel mixed-methods design^30^ combining a national mapping survey (administered via an online questionnaire), longitudinal narrative interviews, ethnographic methods (comprising observations, interviews and document analysis), experience-based co-design (to facilitate the co-production of outputs) and feasibility assessment (to inform translation of findings into practice). Data collection activities, including the survey, interviews with carers and ethnographic observations, began in June 2025 and are currently underway. No analysis has yet been conducted. This multi-methodological approach will enable the generation of rich, contextual data grounded in diverse perspectives, with the overarching aim of informing improvements to the mental healthcare system.^28^

### Research methods

#### Quantitative and mixed-methods

##### National mapping survey (objective 1)

The first component of the study is a multi-site, cross-sectional mixed-methods survey to be undertaken by ward managers of mental health wards providing care for people living with dementia. Our aim is to develop a comprehensive map of care provision across the UK; although we approximate there are at least 100 of these wards—including both NHS and private providers—to date, no complete directory exists.

To map care provision, the survey asks questions around patient profiles (eg, age, gender, admission route), staffing profiles and the ward itself (eg, number of beds, location, type). In line with our research question, there is a particular focus on how wards currently support carers and families of inpatients; specifically, around how carers are involved in care planning, in decisions about their friend or relative, the needs of carers and what ongoing support is offered to them by the ward.

The questionnaire combines both multiple choice and open response items and was co-developed with our PPI advisory group. It is to be completed via an online survey tool which integrates with NHS systems and will not take more than 15 min, recognising limited time and competing demands for managers on these busy wards.

###### Recruitment

Wards are eligible if they deliver care only for people living with dementia or are a mixed older persons’ ward caring for people including those living with dementia. Target recruitment will be 45–60%, in line with NHS workforce surveys.^31^

We will identify mental health wards across the UK via various means, to ensure comprehensive representation. This will include searching NHS Trust, Health Board websites and Care Quality Commission (CQC) reports, contacting members of our national Inpatient Dementia Community of Practice, presenting the study to The Royal College of Psychiatrists Network for Older Adults Mental Health Wards, collaborating with the members of the FIND ME study advisory committee to identify private hospital providers, contacting the executive committee of the Faculty of Old Age Psychiatry and the inpatient workstream of the Faculty of the Psychology of Older People.

###### Analysis

Quantitative data will be analysed using descriptive statistics to provide a summary of responses, and thematic analysis will be conducted of free-text responses.

### Qualitative methods

We will conduct in-depth qualitative research across three NHS mental health wards. The wards will be purposively selected to capture a range of demographic and service contexts, including inner-city, coastal, rural and semi-rural areas with ageing populations and complex health and social care needs. This component comprises two interrelated strands; by combining narrative interviews with ethnographic inquiry, the qualitative work will generate a detailed understanding of carers’ experiences and the organisational factors which shape them. The findings will inform the development of strategies to improve support for carers and enhance outcomes for people living with dementia during mental health ward admissions.

#### Narrative interviews (objective 2)

To explore carers’ experiences, perspectives and support needs across the trajectory of a mental health ward admission, including transition and post-discharge, we will conduct a series of narrative interviews. Where possible, interviews will also include the person living with dementia to ensure their experiences are represented.

##### Longitudinal interviews with current carers

We will recruit 24 carers of people living with dementia currently admitted to a mental health ward (eight per site) to take part in three interviews at 4-month intervals over a 12-month period (total: 72 interviews). Interviews will take place both during the admission and following discharge, to provide insight into how carers’ needs and experiences change over time.

##### Interviews with carers post-discharge

An additional cohort of 24 carers (eight per site) will be recruited, each having supported a person living with dementia discharged after a ward episode within the past 3 years. These one-off, in-depth interviews (total: 24) will enable reflective accounts of care and support needs beyond the immediate crisis period and offer insight into longer-term challenges.

##### Rationale for a longitudinal design

Attempting to fully understand someone’s experience through a single encounter risks incomplete insight and understanding.^32^ A longitudinal design allows: (1) carers to articulate their experiences over time, facilitating the collection of detailed, nuanced data^33^; (2) trust and rapport to be fostered between carers, people living with dementia and the research team, of particular importance when exploring sensitive and emotionally complex issues; (3) alleviated pressures that can arise in single-point interviews, where carers may feel constrained or overwhelmed; and (4) carers to feel empowered and validated; particularly those who may feel marginalised by their experiences, by offering sufficient time and space to share their narratives.^34^

##### Interview methodology

We will adopt a narrative interview approach, encouraging participants to share their personal stories and lived experiences.^35 36^

This methodology is particularly suited to the sensitive nature of admissions to mental health wards and is well-established in research both with individuals living with dementia^37 38^ and their carers.^39 40^ Coming from a position of ‘not knowing’ (Charlés, 2007),^41^ researchers will ask open and prompting questions, allowing participants to direct the conversation and share their experiences.^41^ Guided by our family systems approach (Patterson, 1988),^29^ these interviews will explore how families adapt over time to the stresses of dementia and acute mental health crises.^29^

Each interview will last approximately 60 min and will be arranged at the participant’s convenience. Interviews may be conducted in person (in family/meeting rooms on the ward or in participants’ homes), via video call, or by telephone, according to participant preference. All interviews will be audio-recorded and transcribed verbatim.

##### Inclusive approaches to communication

To promote equity, diversity and inclusion (EDI), we will offer alternative, creative modes of participation to support carers, families and people living with dementia for whom traditional face-to-face interviews may be challenging. All participants will be offered the opportunity to engage through more visual methods, enabling individuals to communicate their experiences in a more accessible and personally meaningful way.^42^

Participants will be invited to take part in an ongoing, creative dialogue over a 12-month period with our experienced artist-researcher. They will receive art materials and pre-paid envelopes to allow them to create and send creative works that express their thoughts and experiences. The artist-researcher will then respond with an artwork of their own, establishing a reciprocal and evolving exchange. This process will continue at a pace set by the participant, allowing them to shape both the content and the duration of the engagement. This inclusive method supports non-verbal expression and provides an important alternative route for participation, to ensure that a wide range of voices and experiences are represented in the study.

##### Recruitment

We will apply purposive sampling to reflect the demographic and cultural characteristics of each site. As the study progresses, this will evolve to ensure the inclusion of carers from diverse backgrounds and family structures. Snowball sampling will also be used to identify additional family members involved in caregiving and decision-making; an approach which has been used effectively in previous studies.^43^

##### Longitudinal interviews with current carers

*Inclusion criteria:* Family member or friend of a person living with dementia currently detained in the ward, aged 18+, able to provide informed consent and willing to participate in interviews.

*Exclusion criteria:* Carers actively involved in safeguarding investigations.

##### Interviews with carers post-discharge

Ward managers will screen electronic or paper records to identify eligible carers. These individuals will receive a letter and Participant Information Sheet (PIS) by post, with translated materials available as needed.

*Inclusion criteria:* Family member or friend of a person living with dementia who has been discharged from the ward within the past 3 years, aged 18+, able to give informed consent and willing to be interviewed.

*Exclusion criteria:* Carers currently involved in safeguarding investigations, or those engaged in ongoing formal complaints or disputes with the Trust.

##### Interviews with people living with dementia

Although many people living with dementia within these wards are in advanced stages of dementia,^44^ some may wish to participate in a joint (dyadic) interview with their carer. Potential participants will be identified through discussions with their carers. Where appropriate, the researcher will meet with the person living with dementia to share a simplified PIS and answer questions.

Capacity to consent will be assessed by the researcher in accordance with the Mental Capacity Act (2005).^45^ This will involve evaluating the participant’s ability to understand, retain and weigh the information and to communicate their decision. If the individual is deemed to have capacity and is willing to participate, a time and date for the interview will be agreed. Consent will be considered an ongoing process, continuously monitored and re-established throughout participation. Capacity will be reassessed before each interview, with written consent obtained using an easy-read consent form. If a person living with dementia loses capacity between interviews, they will not participate in subsequent data collection. During interviews, researchers will remain vigilant for signs of fatigue, distress or withdrawal of consent.

*Inclusion criteria:* People living with dementia currently detained in, or discharged within the last 3 years from, a participating mental health ward; capable of giving informed consent and participating in an interview alongside their carer.

*Exclusion criteria:* Individuals without capacity to consent, or who are involved in safeguarding investigations.

##### Analysis

We will adopt a flexible, iterative approach to data collection and analysis, drawing on theoretical sampling and constant comparison techniques of grounded theory.^46 47^ Survey data, narrative interviews and observations will be collected and analysed concurrently, to inform one another throughout the study. This will enhance the ‘analytic incisiveness’ of the study,^48^ allowing emerging findings to shape subsequent phases of data collection. Narrative analysis of the interviews will ensure that the integrity of individual accounts is retained, preserving the structure, context and meaning of each participant’s story.

A subsequent thematic analysis will be conducted across all interviews to identify recurring patterns and shared meanings across participants and sites.^49^ Coding will focus on the content and meaning of participants’ experiences, with an emphasis on interpreting how people make sense of their interactions with, and transitions between, services.^35^ We aim to generate typologies of experiences and identify factors which support resilience, recovery and adaptation and explore how these processes can be better supported in practice.

##### Artistic dialogues

Artworks will not be analysed as data. Instead, with participant permission, they will be used to support and illustrate the dissemination of findings.

### Ethnographic data collection (objective 3)

To understand how carers are supported and involved in care processes and decisions within mental health wards, we will undertake ethnographic fieldwork within the three wards, incorporating non-participant observation, in situ interviews with staff and carers and analysis of documents routinely shared with carers.

Ethnography enables us to obtain a deep understanding of everyday practices, routines, relational interactions and institutional norms by gathering data from multiple sources.^50 51^ This approach is particularly valuable in healthcare settings where informal, tacit and often unspoken practices shape care experiences^52 53^.

Our ethnographic data collection will seek to understand: (1) ward routines and practices that shape how carers are involved, or excluded, in care decisions and delivery; (2) staff understandings of carer roles, needs and input during the inpatient admission and (3) formal policies and informal rationales that influence carer involvement in decision-making, care provision and discharge planning. This comprehensive approach will provide insight into ward cultures and the systemic and interrelated factors that enable or hinder meaningful carer involvement in care.

#### Methods

We will spend 30 days over a period of 90 days on each of the three wards. An additional fourth month will be dedicated to data cleaning, preliminary analysis and follow-up interviews. The research team will shadow ward staff during routine activities, capturing how care is organised and delivered and how carers are engaged in practice.

#### Observations

Observations will focus on:

- How staff communicate with carers at admission, during visits and through remote updates.
- Handovers, multidisciplinary team meetings and discussions, to observe how carers are considered in planning and decision-making.
- Carer involvement in daily care (eg, personal care, mealtimes), care planning and discharge discussions.

#### In-situ and longer interviews

Short, informal ethnographic interviews (<10 min) will be conducted with staff during shifts to capture real-time reflections on how they recognise carer needs, and the implicit and explicit rationales that shape their decision-making. These will be complemented by longer semi-structured interviews (30–60 min) with five staff per site (total n=15), including ward managers, senior nurses, allied health professionals and consultant psychiatrists.

#### Documentary analysis

We will review documents routinely provided to carers including welcome packs, information leaflets and templates used for recording and sharing information with carers.

#### Recruitment

*Ward staff: *At the start of each observation period, verbal consent will be obtained from all clinical staff working in the areas to be observed. Written consent will be sought at a time that is most convenient for each staff member, before, during or after observation periods, in a manner that avoids disrupting clinical duties.

In-situ interviews: Verbal consent will be taken at the time of the interview, with written consent obtained later when convenient to avoid disrupting care provision.

Longer interviews: Participants will be identified in collaboration with ward managers to ensure a range of professional roles and grades are included. The focus will be on staff whose roles involve regular engagement with families.

*Inclusion criteria:* Staff currently working on one of the three research sites, any professional role with carer contact (eg, healthcare assistant, nurse, occupational therapist, psychologist).

*Carers and people with dementia:* Observations will focus on staff practice and will take place in communal areas (eg, corridors, nursing stations, ward observation points), not at the bedside. While the focus is on staff behaviours, patients may be present in observational spaces. Where patients with dementia are able to consent to being in areas where researchers are conducting observations, this will be sought directly before the study begins. If they lack capacity, we will follow Mental Capacity Act procedures, seeking advice and consent from a personal consultee (typically a family member) or a nominated consultee (eg, the patient’s medical consultant).

No patient will be recruited if the researcher believes participation could negatively impact their mental health or care. If a patient consents but later loses capacity, we will seek consultee advice; if this is not provided or consent is declined, the individual will be withdrawn, and observations will not continue in their presence or about their care.

Verbal consent will be obtained from carers on entry to the ward, and consent will remain ongoing and can be withdrawn at any time. If a carer chooses not to participate, researchers will avoid observation in the area where that carer is present. For prolonged or more focused observations (eg, during multi-disciplinary or discharge planning meetings), written consent will be sought from the carer in advance.

#### Analysis

Initial coding will generate a set of sensitising concepts^54^ that will guide deeper interpretation. These early conceptual insights will serve as a foundation for developing more refined and stable analytic categories. Through an iterative process of comparison and refinement, we aim to move from localised findings to broader conceptual understandings that highlight the structural and systemic conditions shaping experiences within mental health wards.^46^ We will triangulate the survey responses, interview and observation data to enhance the rigour, depth and credibility of our findings.^55^ Triangulation will involve comparing and synthesising the perspectives of people living with dementia, carers and staff to gain a more comprehensive and multi-layered understanding of their experiences. Insights from this component will inform our co-design activities and contribute to a broader understanding of how mental health wards can become more inclusive and supportive of families and carers.

### Experience-based co-design (objective 4)

To translate our research findings into practical, evidence-based strategies, we will use Experience-Based Co-Design (EBCD)^56^ to actively involve people living with dementia, family carers and practitioners in shaping service change. EBCD places lived experience at the centre of service design, supporting the development of relevant, effective and sustainable solutions by incorporating multiple perspectives to meet user needs. EBCD has been successfully used both with people living with dementia and in adult mental health settings.^57^

Drawing on the survey, interview and ethnographic fieldwork data, we will co-produce a series of scenario-based narratives and short films with our PPI Advisory Group. These resources will be grounded in the research and represent composite characters and journeys, to reflect real-life scenarios. The materials will bring the realities of current practice to life, enabling participants to engage with emotionally resonant examples that illustrate how care is currently delivered, and how it might be improved.

#### Workshops

We will conduct a structured series of online workshops, to maximise inclusion of participants from across the UK. Each of three participant groups, people living with dementia (n=10), carers (n=10), and ward staff (n=10), will take part in three 90 min workshops:

1. *Workshop round one—identifying priorities:* Peer-homogenous workshops (one each for people living with dementia, carers and staff) will explore the trigger films and scenario-based materials to surface reflections and identify priority issues in current practice.
2. *Workshop round two—exploring change:* The same groups will reconvene approximately 2 weeks later to focus on opportunities for change. Participants will be encouraged to reflect on what improvements could look like and the groups will co-develop potential strategies for addressing the challenges identified in the first workshop.
3. *Workshop round three—co-design and consensus building:* The final workshops will bring together the three into mixed stakeholder groups (people living with dementia, carers and staff, 10 participants in each workshop) to collaboratively develop and refine a provisional model of best practice and identify practical strategies and resources to support change on mental health wards. These sessions will prioritise consensus-building across perspectives and professional boundaries.

#### Recruitment

*People living with dementia and carers (n=10 each*): Participants will be recruited through national partners: Dementia UK, Young Dementia Network, John’s Campaign and Together in Dementia Everyday (TiDE).

*Inclusion criteria:* People living with dementia or carers, aged 18+, able to provide written informed consent, willing to discuss mental health and dementia, able to participate in online workshops

Purposive sampling will ensure diversity of experience and alignment with EDI objectives. Written consent will be obtained before the first workshop, and verbal consent will be reconfirmed at subsequent workshops.

*Mental health ward staff (n=10):* Staff will be recruited via the Royal College of Psychiatrists’ accreditation network, the inpatient dementia community of practice and study newsletter mailing lists. A recruitment poster will be distributed through emails, newsletters and shared on LinkedIn. Purposive sampling will ensure representation across disciplines, bands and levels of experience.

*Inclusion criteria: s*taff currently working on a mental health ward which cares for people living with dementia, any professional role with carer contact (eg, healthcare assistant, nurse, occupational therapist, psychologist or psychiatrist), employed in NHS or private sector.

*Exclusion criteria:* staff currently involved in an active safeguarding investigation.

#### Analysis

We will use thematic analysis^49^ to explore patterns and recurrent narratives in the workshop transcripts. This approach will allow us to capture the complexity and nuance of participants’ experiences and enhance the ‘analytic incisiveness’ of our findings.^58^ Insights from the first two rounds of workshops will inform the development of a provisional model of best practice for supporting and partnering with carers in mental health wards. This model, along with practical evidence-based strategies for implementation on the wards, will be refined through consultation in the third round of mixed-stakeholder workshops.

### Feasibility study (objective 5)

Finally, we will conduct a feasibility study of implementing the co-designed strategies to facilitate carer involvement in admission of their friend or relative in practice. Implementation outcomes must be considered to evaluate their feasibility. Outcomes can be separated into those which assess the potential effects of implementation strategies: adoption, fidelity (adherence), penetration (reach) and sustainability (maintenance), and those which inform the development of the implementation strategies: acceptability, feasibility, appropriateness and cost.^59^

As this is a preliminary feasibility exploration ahead of potential roll-out in practice, and due to the discrete length of the study, we will focus only on those which inform the further refinement and development of the strategies and resources.

Qualitative methods will be used to assess (1) acceptability, whether participants perceive the strategies to be agreeable and acceptable in terms of their content, delivery and complexity and (2) feasibility, the extent to which participants perceive the strategies can be successfully used and integrated into routine care.

#### Focus groups with staff (n=30)

Three online focus groups (8–10 participants each) will be held with mental health ward staff from across the UK. 2 weeks prior to the focus groups, outputs from the co-design study will be circulated for participant review. Focus groups will involve discussions around what factors might facilitate implementation of the materials within the mental health ward setting, barriers which might impede uptake and what strategies could be employed to overcome them.

#### Feedback from people living with dementia and carers (n=30 each)

People living with dementia and carers will be invited to provide feedback on the strategies and materials in their preferred format by email, phone, video call or through annotated documents. The research team will also collect group feedback from the FIND ME Study PPI Advisory Group.

*Recruitment of people living with dementia and carers (n=30 each):* Participants will be recruited through our national partners—Dementia UK, TiDE and John’s Campaign—via posters shared through their PPI groups, newsletters and social media. To support inclusion of ethnic minority groups, we will also distribute materials through faith and community organisations.

*Inclusion criteria:* People living with dementia or carers (current or bereaved), aged 18+, willing to review materials related to mental health wards.

*Exclusion criteria*: Involvement in an active safeguarding investigation.

*Mental health ward staff (n=30):* Staff will be recruited via the inpatient dementia community of practice mailing list, the Royal College of Psychiatrists’ list of accredited dementia mental health wards and wards who participated in our survey.

*Inclusion criteria*: staff from any professional group working in mental health wards for people living with dementia, including students and staff from any band/grade, on NHS or private wards, in roles involving carer contact.

*Exclusion criteria:* active involvement in a safeguarding investigation.

#### Analysis

Focus group transcripts will be analysed inductively. Feedback related to specific changes to the documents (eg, wording, tone or format) will be coded and documented separately for research team review. Insights from the thematic analysis will be used to refine and improve the materials developed, to enhance their suitability for ward use.

### Ethics and dissemination

Ethical approval has been granted by London Camberwell St Giles Research Ethics Committee and the Health Research Authority (REC Ref: 25/LO/0040). Informed consent will be obtained from all participants, with capacity assessed in line with the Mental Capacity Act (2005).

### Outputs

The project will generate a set of practical, accessible resources to support mental health ward staff, carers and people living with dementia. These will include:

- Broadcast-quality podcasts, short films, audiobooks and downloadable guides detailing best practice, delivery of culturally appropriate care and recognising carer needs.
- Staff-facing training materials focused on effective carer engagement and navigating key points in the care pathway.
- Guides for carers explaining mental health ward processes, legal rights and discharge preparation.
- Easy-read and accessible resources for people living with dementia.

All materials will be freely available in multiple formats and languages (including Arabic, Bengali, British Sign Language, Chinese, Gujarati, Hindi, Polish, Punjabi, Sylheti and Urdu) and distributed via widely used platforms such as Spotify, YouTube, Apple/Google Podcasts and partner websites including Dementia UK and collaborating academic institutions.

### Dissemination Strategy

A national dissemination strategy will ensure wide reach and uptake of the outputs. This will be facilitated through:

- Our mapping survey to engage wards nationally, support dissemination and monitor reach.
- Targeted communication with mental health wards via newsletters and training events.
- Rapid sharing of findings through online masterclasses, conferences and workshops.
- Strategic partnerships with key stakeholders, including the Royal College of Psychiatrists, the Inpatient Dementia Community of Practice and the Faculty of the Psychology of Older People.

Members of the project’s steering group—including NHS England, Dementia UK and John’s Campaign—will play an active role in promoting the resources and supporting their integration into policy and practice nationally.
