# Supplementary material for: Family interventions in dementia mental health environments (FIND ME): a mixed-methods protocol
Source: BMJ Open. 2026 Jan 28;16(1):e110244. doi: 10.1136/bmjopen-2025-110244 (PMC12853441; doi:10.1136/bmjopen-2025-110244)
Supplement: Supplementary file 1 [file bmjopen-2025-110244.reviewer_comments.pdf]

PEER REVIEW HISTORY

BMJ Open publishes all reviews undertaken for accepted manuscripts. Reviewers are asked to complete a checklist review form and are provided with free text boxes to elaborate on their assessment. These free text comments are reproduced below.

ARTICLE DETAILS

Title (Provisional)

Family Interventions in Dementia Mental Health Environments (FIND ME): A mixed-methods protocol

Authors

Wolverson, Emma; Gillam, Juliet; Dunn, Rosie; Hoe, Juanita; Underwood, Ben R.; Mwale, Shadreck; Credland, Nicki; Robertson, Martin; Wyatt, Megan; Lane, Rhiannon; Featherstone, Katie

---

VERSION 1 - REVIEW

**Reviewer**	**1**
**Name**	**Laver, Kate**
**Affiliation**	**Flinders University, Flinders University, Department of Rehabilitation**
**Date**	**29-Nov-2025**
**COI**	**None**

I have provided some comments which I hope will help the authors improve the paper.

Overall I would recommend a bit more editing to the intro. There are many small paragraphs and flow and 'setting the scene' could be much improved.

1. The first paragraph seems a bit misaligned with the focus of the project which relates more to BPSD and mental health and family rather than physical symptoms.

2. The second paragraph I feel must be UK specific. In other countries (eg Australia), people with dementia tend to only be admitted to mental health wards when they have mental health comorbidities rather than just changes in mood or behaviour. I feel like this paragraph is a bit broad at the moment.

3 Intro: you state that 'sufficient carer invovement directly improves outcomes for patients residing on mental health wards" - can you be a bit more specific about this research? (eg are there many studies? what design? what outcomes?)

4. It would help to be clearer about the primary intended outcome - is this to improve family and carer outcomes or to improve outcomes for the person with dementia? or quality of care?

Overall the methods are well described.

VERSION 1 - AUTHOR RESPONSE

Reviewer: 1
Dr. Kate Laver, Flinders University
Comments to the Author:
I have provided some comments which I hope will help the authors improve the paper. Overall I would recommend a bit more editing to the intro. There are many small paragraphs and flow and 'setting the scene' could be much improved.
1. The first paragraph seems a bit misaligned with the focus of the project which relates more to BPSD and mental health and family rather than physical symptoms.
2. The second paragraph I feel must be UK specific. In other countries (eg Australia), people with dementia tend to only be admitted to mental health wards when they have mental health comorbidities rather than just changes in mood or behaviour. I feel like this paragraph is a bit broad at the moment.
3 Intro: you state that 'sufficient carer invovement directly improves outcomes for patients residing on mental health wards" - can you be a bit more specific about this research? (eg are there many studies? what design? what outcomes?)
4. It would help to be clearer about the primary intended outcome - is this to improve family and carer outcomes or to improve outcomes for the person with dementia? or quality of care?
Overall the methods are well described.

1. The first paragraph seems a bit misaligned with the focus of the project which relates more to BPSD and mental health and family rather than physical symptoms.

Thank you for this – agree, have removed the less relevant section and created one more focused paragraph.

2. The second paragraph I feel must be UK specific. In other countries (eg Australia), people with dementia tend to only be admitted to mental health wards when they have mental health comorbidities rather than just changes in mood or behaviour. I feel like this paragraph is a bit broad at the moment.

I have clarified that we are talking about the process in the UK by amending the paragraph to explicitly state it – thank you.

3 Intro: you state that 'sufficient carer involvement directly improves outcomes for patients residing on mental health wards" - can you be a bit more specific about this research? (eg are there many studies? what design? what outcomes?)

Thank you for this. I have elaborated on the statement and added the following:

Previous research with other patient groups has demonstrated that sufficient carer

involvement directly improves outcomes for patients residing on mental health wards. Family interventions - structured, therapeutic programmes designed to improve communication, coping, and collaboration within families - are associated with better symptom control and improved functioning for adults with mental health conditions (Carr, 2009), reduced relapse rates, lower hospital readmissions, and improved medication adherence – all key determinants of long-term stability (Pharoah et al., 2010; Pilling et al., 2002). In addition, the National Institute for Clinical Excellence (NICE) highlights that supported and informed carers contribute to earlier detection of deterioration, improved engagement with treatment and more consistent, coordinated care for patients (National Institute for Clinical Excellence, 2020).

4. It would help to be clearer about the primary intended outcome - is this to improve family and carer outcomes or to improve outcomes for the person with dementia? or quality of care?

Thank you. We hope that the evidence-based strategies we plan to develop will improve both family carer experiences (e.g. support, involvement) when a person living with dementia is admitted to a mental health ward, and also to improve outcomes for which people with dementia receive on a mental health given what we know from previous research on family involvement on patient outcomes (please see above in response to point 3). I have clarified this in the manuscript.
